# CCL2/CCR2 signaling in cancer pathogenesis

**DOI:** 10.1186/s12964-020-00589-8

**Published:** 2020-05-29

**Authors:** Qiongyu Hao, Jaydutt V. Vadgama, Piwen Wang

**Affiliations:** 1 Division of Cancer Research and Training, Charles R. Drew University of Medicine and Science, Los Angeles, CA 90059 USA; 2 David Geffen School of Medicine, University of California, Los Angeles, CA 90095 USA; 3 Center for Human Nutrition, University of California, Los Angeles, CA 90095 USA

## Abstract

Chemokines are a family of small cytokines, which guide a variety of immune/inflammatory cells to the site of tumor in tumorigenesis. A dysregulated expression of chemokines is implicated in different types of cancer including prostate cancer. The progression and metastasis of prostate cancer involve a complex network of chemokines that regulate the recruitment and trafficking of immune cells. The chemokine CCL2 and its main receptor CCR2 have been receiving particular interest on their roles in cancer pathogenesis. The up-regulation of CCL2/CCR2 and varied immune conditions in prostate cancer, are associated with cancer advancement, metastasis, and relapse. Here we reviewed recent findings, which link CCL2/CCR2 to the inflammation and cancer pathogenesis, and discussed the therapeutic potential of CCL2/CCR2 axis in cancer treatment based on results from our group and other investigators, with a major focus on prostate cancer.

Video Abstract

Video Abstract

## Background

Chemokines and cytokines are core regulators in cancer microenvironment which has been established as one of the hallmark drivers of cancer [1]. The cellular composition of tumor microenvironment is frequently modulated by cytokine milieu secreted by cancer cells in favor of tumor progression [1, 2]. Inflammation is one of the initiating processes of carcinogenesis where inflammatory/immune cells are trafficked into the tumor microenvironment by specific cytokines termed chemokines [3]. Chemokines are a family of small cytokines with ability to induce chemotaxis, a process where directed migration of cells expressing the appropriate chemokine receptor occurs towards higher local concentrations of chemokine ligands. Chemokines guide a variety of immune cells to the site of tumor initiation and subsequently lead to an inflammatory/immune response [3]. Chemokines contribute to the development of malignancy through roles in progression, migration, angiogenesis, and metastases in multiple cancer types [4]. Elevated levels of cytokines/chemokines such as IL-8 (interleukin-8), CXCL1 (Chemokine (C-X-C motif) Ligand 1), CCL2 (Chemokine (C-C motif) ligand 2, also known as monocyte chemoattractant protein-1, MCP-1), and CXCL5 have been associated with increased growth and progression of breast, ovarian, and prostate cancer [5–9]. In addition to tumor cells, various cells in the host microenvironment, including infiltrating leukocytes, endothelial cells, and fibroblasts, as well as adipocytes, are able to produce cytokines/chemokines such as CCL2 for tumor growth and progression [4, 10–26].

The upregulation of CCR2 has been found to be associated with advanced cancer, metastasis, and relapse [27]. The overexpression of CCL2 and resultant promotion of tumor growth have also been observed in breast [11, 12], ovarian [13], esophageal [14], gastric [15], renal cell [16], lung [17], colon [18], and papillary thyroid cancers [19]. In breast tumors, CCL2 overexpression was associated with advanced disease, tumor progression, and angiogenesis [20], and predicts prognosis and recurrence [22]. In breast tumor bone metastases, CCL2 overexpression led to enhanced osteolysis and the release of bone matrix-bound angiogenic factors, including platelet-derived growth factor, fibroblast growth factors-1, and transforming growth factor b [21]. Several studies have also demonstrated that serum CCL2 was elevated and associated with tumor stage in patients with breast, ovarian, and lung cancers [23–25].

Prostate cancer (PCa) is one of the most common types of cancer and the second leading cause of cancer death in men in the United States [28]. The morbidity of PCa has still been increasing among elderly men over the last decade [29]. PCa progression and metastasis is driven by many factors including the abnormalities of many growth factors and cytokines, among others such as the mutation and/or amplification of androgen receptor and other oncogenes and the inhibition of tumor suppressor genes [30–32]. The overexpression of CCL2 and its main receptor CCR2 (CC chemokine receptor 2) has been observed in both primary and metastatic PCa cells [33].

In addition, Lu et al. reported that elevated serum CCL2 was associated with bone metastasis in a study of 39 prostate cancer patients at various stages, suggesting the possibility of using serum CCL2 as a prognostic biomarker [26]. Since elevated CCL2 in circulation is also one of the typical features of obesity [34–38], this supports the role of CCL2 in connection of obesity and cancer promotion. These results suggest the critical role of the CCL2-CCR2 axis in cancer progression and its potential use as therapeutic target.

### Classification of chemokines

Chemokines are a family of small chemotactic cytokines, which are signaling proteins secreted by cells. Chemokines have been classified based on the relative position of cysteine residues near the N terminus into four major families: CC, CXC, C, and CX_3_C. The CC chemokine (or β-chemokine) proteins have two adjacent cysteines near their amino terminus. There have been at least 27 distinct members of this subgroup reported for mammals, called CC chemokine ligands (CCL)-1 to − 28. CXC chemokines (or α-chemokines) have two N-terminal cysteines, which are separated by one amino acid, represented in this name with an “X”. There have been 17 different CXC chemokines described in mammals. C chemokines (or γ chemokines), are unlike other chemokines in that they have only two cysteines, one N-terminal cysteine and one cysteine downstream. Two chemokines have been described for this subgroup and are called XCL1 (lymphotactin-α) and XCL2 (lymphotactin-β). CX_3_C chemokines (or d-chemokines) have three amino acids between the two cysteines. The only CX_3_C chemokine discovered to date is called fractalkine (or CX_3_CL1). Chemokine receptors are G-protein coupled receptors located in the cell membrane, and they transduce the extracellular signal by interacting with chemokine ligands [39]. Chemokines have substantial effects as chemotactic factors on normal development, inflammation, atherosclerosis, and angiogenesis [40]. Chemokines have been implicated in many aspects of tumorigenesis, including the regulation of cancer cell growth, angiogenesis, metastasis, and host immune response [41].

### CCL2/CCR2

CCL2 (MCP-1) is a member of the CC chemokine family [42]. The CCL2 gene is located in the q11.2-q12 region of human chromosome 17, and it encodes a precursor protein of 99 amino acids that matures into 75 amino acids in size [43]. CCL2 is initially described as a “tumor-derived chemotactic factor”, and has been shown to be a potent chemoattractant for several types of immune cells, including the monocytes, natural killer cells, memory T cells, and immature dendritic cells, thereby mediating multiple proinflammatory effects and neoangiogenesis [44–50]. CCR2 has been found to be expressed by multiple cell types including monocytes, dendritic cells (DCs), endothelial cells, and cancer cells [51–54]. CCL2 functions through binding to CCR2, one of 19 members of the human chemokine receptor family [55]. In addition to CCL2, CCR2 has several other high-affinity ligands, including CCL7 (MCP-2), CCL8 (MCP-3), CCL13 (MCP-4), and CCL12 (MCP-5) - a murine chemokine with close homology to human CCL2 [56–59], with an order of strength in binding to CCR2 as CCL2 > > CCL13 = CCL8 > CCL7 [60]. Monocytes, immature dendritic cells, and T-cell subpopulations have high expression of CCR2 which mediates their migration towards CC chemokines such as CCL2 [61]. CCL2-CCR2 signaling axis is implicated in many inflammatory and neurodegenerative diseases such as atherosclerosis, multiple sclerosis, asthma, neuropathic pain, diabetic nephropathy, and cancer [62, 63], and therefore is explored as a potential target for the treatment of these diseases.

## The role of CCL2-CCR2 axis involving immune cells in tumor progression

**Fig. 1:**
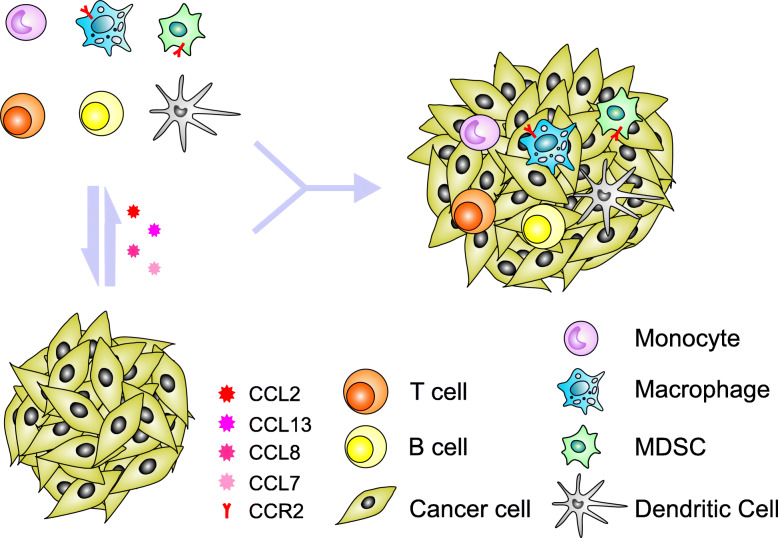
Crosstalk between tumor cells and pro-inflammatory factors in the tumor microenvironment further amplifies the extravasation of tumor cells. Tumor cells produce chemokines, such as CCL2, CCL7, CCL8 and CCL13 that drive the generation of multiple types of regulatory immune cells, including T cells, B cells and myeloid-derived suppressor cells (MDSCs), and promote further TAM development by enhancing the recruitment of macrophages to the tumor site. Immune Cells in the modified tumor microenvironment subsequently produce cytokines, chemokines and other molecules that promote immune escape of tumor cells

The inflammatory tumor microenvironment includes diverse host cells that are chemoattracted and induced by tumor-produced factors to generate a highly immune suppressive environment (Fig. 1), in which cross-talk between macrophages, myeloid-derived suppressor cells (MDSCs), and dendritic cells (DCs) reduces the opportunity to activate tumor-reactive T cells and thereby provides an environment for immune escape and continued tumor progression [64, 65].

### Myeloid innate immune cells and myeloid-derived suppressor cells (MDSCs)

Monocytes, macrophages, and DCs are a heterogenous population of myeloid innate immune cells characterized by phagocytic and antigen-presenting capacities [66]. Monocytes circulate in the bloodstream for about 1 to 3 days and then typically extravasate into tissues where they differentiate into tissue resident macrophages or DCs, controlled by local environmental signals, such as colony-stimulating factor-1 (CSF-1) [67, 68].

Macrophages are a population of terminally differentiated myeloid cells and tissue-resident cells derived from monocytes circulating in peripheral blood [69]. Macrophages promote host survival by regulating adaptive immunity, eliminating infectious agents, and promoting wound healing in healthy individuals [70]. Extensive literature demonstrates that macrophages are co-opted to facilitate tumor growth during malignancy in both mice and humans [71–74]. The macrophage population is comprised of a continuous spectrum of phenotypically distinct subpopulations in their tissue microenvironment, demonstrating the complexity of this cell population [75]. The terminology ‘M1 macrophage’ and ‘M2 macrophage’ was coined to describe the different functional states of macrophages [76]. M1 or ‘classically activated’ macrophages are tumoricidal. They are activated by IFNγ or lipopolysaccharide, and characterized by high expression of IL-12 (Interleukin-12) and low expression of IL-10 (Interleukin-10). Under normal conditions, M1 macrophages are responsible for protecting the host against infection and injury and facilitating tissue remodeling [68], also suspected to be important in the formation of important organs like the heart and brain [77]. In contrast, M2 macrophages are activated by IL-4, IL-13, IL-10, and glucocorticoid hormones, produce high levels of IL-10 and low levels of IL-12, and promote tumor progression [26]. Macrophage phenotype driven by the local tumor microenvironment strongly polarizes towards an M2-like phenotype, giving rise to so-called “tumor associated macrophages” (TAMs) [78, 79]. However, studies have shown that a small number of TAM express both M1 and M2 markers [67]. The exclusive characterization of macrophage populations into M1 and M2 subtypes could be excessively simplistic, as macrophages have been found to be highly plastic cells that can demonstrate a variety of phenotypes [80]. Thus, a more comprehensive classification of TAM is needed which requires the integration of a multiparameter analysis of cell surface markers, comparison of TAM transcriptome, and consideration of the dynamic nature of macrophages [81].

TAMs enhance malignancy and promote numerous important features of tumor progression via both non-immune and immune mechanisms, including angiogenesis, motility, metastasis, and inhibition of T cell function [71]. Additionally, TAMs are also known to suppress responses to therapeutics, including chemotherapy, irradiation and angiogenic inhibitors [71, 82, 83]. In addition to malignancies, macrophages are associated with the progression of a number of other diseases such as asthma, allergic inflammation, and rheumatic inflammatory diseases [84, 85].

MDSCs are immune suppressive immature myeloid cells that are virtually elevated in all patients and experimental mice with malignancies [64]. MDSCs comprise a heterogeneous population of immature myeloid cells from the myeloid lineage characterized by co-expression of CD11b and Gr-1 and lack features of mature macrophages and dendritic cells in tumor-bearing mice. MDSCs can be divided into two distinct sub-populations: monocytic MDSCs (Mo-MDSCs) and polymorphonuclear (PMN)-MDSCs, also known as granulocytic (G)-MDSCs [75]. These two subsets differ in their gene expression profiles and immunosuppressive activities [86]. MDSCs strongly expand in pathological situations such as chronic infections and cancer, as a result of an altered haematopoiesis [86]. Marvel et al. found that activation of the immature myeloid cells via a network of regulatory mechanisms results in the accumulation of MDSCs in mice with transplantable and spontaneous tumors [87, 88]. MDSCs are discriminated from other myeloid cell types in which they possess strong immunosuppressive activities rather than immunostimulatory properties. MDSCs interact with other immune cell types including T cells, dendritic cells, macrophages and natural killer cells to regulate their functions. Accumulating evidence supports that MDSCs contribute to cancer immune evasion and tumor growth by suppressing T cell anti-tumor functions and modulating innate immune responses [86] as well as through non-immune suppressive pathway [89, 90]. MDSCs also accelerate angiogenesis, and subsequently promote tumor progression and metastasis through the expression of cytokines [91]. In many cancers, blood MDSC numbers correlate with stage and metastatic burden [92].

MDSCs and macrophages, two myeloid derived populations, are inextricably interconnected in the tumor microenvironment. They directly impact each other in a reciprocal fashion via the production of IL-10 and IL-6, respectively. Induction of one population favors the development of the other population. Clinical and experimental evidence has shown that cancer tissues with high infiltration of MDSCs and TAM are associated with poor patient prognosis and resistance to therapies [93–95]. Therefore, they have become a key therapeutic target.

### CCL2-CCR2 axis in recruitment of monocytes and macrophages to tumor sites

Many types of cells present in the primary and metastatic tumor microenvironments, including stromal cells, leukocytes, endothelial cells, and tumor cells, produce CCL2 [11]. Prostate cancer cells LNCaP, C4-2B, PC-3, and VCaP produce higher amounts of CCL2 than primary prostate epithelial cells [96]. Tumor and stroma cells secrete CCL2 to recruit inflammatory monocytes and TAMs expressing CCR2 [2]. Monocytes recruited to tumors sites through the CCL2-CCR2 axis are polarized to TAMs, contributing to tumor cell survival [97]. Two prior studies from McClellan and Popivanova et al. suggested that CCL2 increased colon tumor numbers in mice through a CCL2-CCR2 dependent recruitment of myeloid cells [98]. Inhibition of CCL2-CCR2 signaling blocks the recruitment of inflammatory immune cells, and inhibits cancer cells metastasis in tumor-bearing mice [34–37, 63, 99–104].

Macrophage composition in different tissues or inflammatory environments depends on a dynamic equilibrium between recruited and tissue-resident macrophages [105]. Macrophages in the colonic mucosa are derived from circulating Ly6C^+^CCR2^+^ monocytes, during inflammation and under steady-state conditions [106]. In cancer, the evidence to date indicates that TAMs are dynamically replaced by circulating precursors. Both the tissue resident macrophages present in normal mammary tissues and TAMs that develop during tumor progression in the MMTV-PyMT breast cancer model are derived from blood-circulating CCR2^+^ monocytes, but only TAMs display self-renewal capability [107]. Elevated number of circulating blood monocytes and high macrophage infiltration into tumor tissues have been associated with poor clinical outcome in patients with various cancer types [2, 22, 71, 76, 82, 83, 108–113]. Hence, therapeutic strategies that either target TAM recruitment from inflammatory monocytes, or deplete TAMs will benefit patients with cancer or inflammatory diseases [114].

### CCL2-CCR2 axis involving MDSCs in tumor progression

Overall, factors regulating MDSC accumulation and mechanisms of MDSCs’ action in cancer promotion remain underexplored. Several studies have demonstrated a role of CCL2 in recruiting MDSCs to tumor sites. Using human colorectal cancer (CRC) samples in conjunction with mouse models of colorectal carcinogenesis, Chun et al. identified a pro-neoplastic role for CCL2 in influencing MDSC accumulation and importance of MDSCs and CCL2 in tumor microenvironment during the development of CRC [115]. CCL2 and GM-CSF (Granulocyte-macrophage colony-stimulating factor) induced by oncogenic fusion protein RET/PTC3 together promote the recruitment of CD11b^+^GR1^+^ MDSCs that can promote thyroid carcinomas progression [116, 117]. Moreover, the formation of invasive squamous cell cancer and the associated production of CCL2, GM-CSF, M-CSF (Macrophage colony-stimulating factor) and TNF (Tumor Necrosis Factor) caused by conditional deletion of the gene encoding p120 catenin in mice resulted in the accumulation of immunosuppressive CD11b^+^GR1^+^CD124^+^ MDSCs, which activated stromal fibroblasts and promoted tumor progression [118]. Although TAMs and MDSCs are regarded as separate entities, the boundaries between them are not clearly demarcated, and they share many characteristics [119]. Intriguingly, while PMN-MDSCs increased in castrated tumors models of prostate cancer (TRAMP-C1 and MyC-CaP), the frequency of tumor infiltrating macrophages (TAMs) decreased [120], suggesting that MDSCs confers more profound suppression on the immune cells in prostatic tumor microenvironment.

## The role of CCL2-CCR2 axis in prostate cancer progression

PCa is the fifth leading cause of cancer death in men worldwide. The development and progression of PCa is typically associated with an inflammatory microenvironment [121]. The involvement of CCL2-CCR2 axis in PCa progression has been consistently observed in many studies, including an enhanced CCL2-CCR2 signaling and tumor promotion under obese conditions. Therefore, PCa seems to be a good example to demonstrate the role of CCL2-CCR2 in connection of inflammation/obesity to tumor pathogenesis.

### Overview of inflammation and obesity-induced inflammation in prostate cancer

Inflammation is a complex biological process and a protective response of the immune system to establish a physical barrier against the harmful stimuli, such as infection or irritation, involving molecular mediators, immune cells, and blood vessels [122]. Inflammatory cells consist of lymphocytes, neutrophils, eosinophils, plasma cells, and histiocytes, which are innate immune cells playing a major role in inflammatory process. The presence of inflammatory cells does not signify the cells themselves are inflammatory. A more exact designation is “cells entering inflammatory tissue”. Inflammation can be either acute or chronic. Acute inflammation is an immediate and innate response to harmful stimuli and efficiently minimizes impending injury by molecular, cellular events and interactions. Chronic inflammation is derived from uncontrolled acute inflammation, also known as prolonged inflammation, which is associated with various diseases, such as type 2 diabetes, atherosclerosis, rheumatoid arthritis, asthma, and cancer [123]. Currently, chronic inflammation is estimated to account for approximately 15 to 25% of human cancers [124–126]. Nearly all primary malignant neoplasms are associated with dense infiltrates of inflammatory cells. Macrophages, neutrophils and lymphocytes are the most abundant immune cells in the tumor microenvironment [95]. In the tumor microenvironments, the interactions among cancer cells, immune cells, endothelial cells, and fibroblasts can play important roles to contribute to tumor progression.

Obesity is characterized by an excess of body fat resulting from a chronic positive energy balance. Obesity has been associated with increased risk for metabolic diseases and cancers such as esophagus, gastric, breast, pancreas, colon, liver, endometrial, kidney, and prostate cancer [127–129]. In PCa, prospective studies in the United States showed that body mass index (BMI) was weakly and positively associated with PCa, and greater BMI was an independent predictor of PCa [130, 131]. Higher BMI was associated with biochemical recurrence of PCa after radical prostatectomy in an analysis of 4123 men treated by radical prostatectomy [132], and higher BMI was positivity correlated with the PCa death in a prospective study of 404,576 men [128]. A meta-analysis of advanced PCa showed a positive linear relationship with BMI for advanced PCa [133]. These epidemiological studies have shown consistent evidence of the association of obesity with advance PCa.

Adipose tissue is mainly composed of adipocytes, while stromal vascular fraction (SVF) including adipocyte derived stem cells, preadipocytes, lymphocytes, macrophages, fibroblasts and vascular endothelial cells also contribute to the growth and function of adipose tissue [134, 135]. Mature adipocytes have been considered not only as energy-storing cells, but also as highly endocrine cells which are able to secrete an heterogeneous group of molecules termed ‘adipokines’ such as chemokines, growth factors, hormones, or pro-inflammatory molecules [134, 136].

Obesity is associated with a chronic low-grade systemic inflammation that has been implicated in the development of common, medically important complications, including atherosclerosis, hepatic steatosis, and insulin resistance [137–140]. One characteristic of obesity-caused inflammation is the activation of pathways that regulate inflammation, such as JNK and NF-κB pathways [141–143], and immune cells infiltrating in the white adipose tissue [144–147]. Activation of these cells elevates local and systemic expression of pro-inflammatory molecules, including acute-phase reactants, procoagulant factors, chemokines, and cytokines (such as TNF, HMGB1, IL-1, and IL-6), and mediates the inflammatory response [123]. Elevated cytokine and chemokine levels are typically associated with obesity and propagate the obesity-associated inflammatory state [148–151]. Obesity also causes the accumulation of macrophages in adipose tissue [100], which have been implicated in the development and maintenance of obesity-induced adipose tissue inflammation [121, 152].

Obesity-induced inflammatory state contributes to PCa development [99]. T cells are accumulated in prostate tumor of a diet-induced obese Hi-Myc mice [153]. The cytotoxic function of NK cells to PCa cells is inhibited by humoral factors from adipocytes [154]. Myeloid differentiation is skewed towards the expansion of MDSCs under chronic inflammatory conditions or cancer [86]. These MDSCs infiltrate inflammation sites and tumors, where they stop immune responses by inhibiting T cells and NK cells. Other inflammatory cells and immune cells could be also involved in the PCa progression. These local inflammatory cells orchestrate an environment that fosters tumor proliferation and survival [155]. In a study of the relationship between inflammation and tumor progression in the prostate, Fujita et al. found that secretion of IL-6 from local macrophages was increased in prostate tissues of HFD-fed mice, and inhibition of the IL-6 pathway resulted in the suppression of tumor growth [156]. Mechanistic studies demonstrated that IL-6 might promote the proliferation of PCa cells via the STAT3 pathways and an increase of local MDSCs [157]. These results suggest that inflammation plays a central role for the progression of PCa in the studied obese state.

### CCL2-CCR2 axis in mediating the interplays among microenvironment, inflammation/obesity, and prostate cancer

The cytokines and chemokines produced by prostatic tumor cells and various cells in the host microenvironment, including infiltrating leukocytes, endothelial cells, and fibroblasts [4, 10], enhance the growth, progression, migration/invasion, and metastasis of prostate cancer [8]. CCL2 has been identified as a prominent modulator in such a dynamic tumor-host interactions [8]. CCL2 was overexpressed in primary prostatic tumors as determined by immunohistochemistry [96]. In advanced prostate cancer, CCL2 expression was also notably higher in the metastatic tumor-bone microenvironment compared with that in bone marrow adjacent to the tumor as measured by cytokine arrays [158]. CCL2 acts in a paracrine and autocrine manner to stimulate PCa cell proliferation and migration. Although the molecular link between CCL2 and PCa has not been thoroughly elucidated, several studies have suggested the involvement of CCR2 in mediating the signaling of CCL2 in PCa progression [54]. High levels of CCR2 exist in prostate tumor cell surface to respond to autocrine and/or paracrine CCL2 in the microenvironment. The mRNA and protein expression of CCR2 were higher in aggressive cell lines such as DU145, PC-3, and C4-2B compared with androgen-sensitive LNCaP cells and non-neoplastic prostate epithelial cells [8, 54], and higher in prostate cancer metastatic tissues as compared with localized prostate cancer and benign prostate tissue [43]. Analysis of real-time PCR and IHC staining on tissue microarray specimens revealed that higher CCR2 expression was also associated with higher Gleason score and higher clinical pathologic stages [54], suggesting a positive association between CCR2 expression and prostate cancer progression [54]. Further, CCL2-induced prostate cancer cell chemotaxis was abolished by a CCR2 antagonist, which confirmed that CCR2 is the functional receptor of CCL2 [96].

The downstream target of CCR2 may include the PI3K/Akt signaling pathway [159]. Upon activation by CCL2-CCR2, PI3K/Akt activates mTORC1 and up-regulates survivin which is a key molecule protecting prostate cancer cells from autophagic death [160, 161]. CCL2 was also able to stimulate prostate cancer cells to extravasate into the bone through a layer of bone marrow endothelial cells partially by the activation of the small GTPase Rac through the actin-associated protein PCNT1 [162]. These observations suggest the role of CCL2 from tumor microenvironment in stimulation of prostate cancer expansion and metastasis [127–129, 134–136].

The prostate gland is surrounded by periprostatic adipose tissue (PPAT) [163]. Excess visceral adiposity around the prostate can lead to changes of the secretory pattern of adipocytes as well as to subsequent modifications in the cellular composition of periprostatic environment [134, 135]. Evidence has shown a correlation between the abundance of PPAT and tumor aggressiveness, suggesting a paracrine role of PPAT during tumorigenesis [164]. Extraprostatic spreading of PCa into PPAT is found to be a more important determinant of cancer recurrence than an invasive phenotype [165, 166]. A high infiltration of macrophages was observed in PPAT in obese animal models [163], which may be due to an increased secretion of CCL2 by adipocytes in the obese conditions. Once recruited in the tumor microenvironment by adipocytes, macrophages tend to have metabolic reprogramming and be polarized into M2 phenotype by tumor cells, favoring tumor growth and progression [167, 168]. Enhanced infiltration of activated macrophages in visceral adipose tissues was also observed in obese patients [100], and CCR2 seemed to have a direct role in the recruitment of macrophages. Ccr2-knockout animals had significantly fewer adipose tissue macrophages than wild-type mice [101]. Both Ccr2 genetic deficiency and pharmacological inhibition reduced macrophage content of adipose tissue, and improved inflammatory profile of adipose tissue including increase in adiponectin expression and amelioration in systemic glucose homeostasis [102].

Studies using high-fat diet (HFD)-induced obese mouse models have also demonstrated the tumor-promoting effect of obesity on prostate cancer. HFD promoted androgen-sensitive PCa growth and progression to androgen-independent growth in mouse models [169, 170]. Conditioned medium using serum collected from HFD-fed TRAMP mice promoted the proliferation, migration, and invasion of DU-145 cells [171]. In a prostate tumor xenograft model of mice implanted with LNCaP cells, HFD promoted tumor growth and increased blood CCL2 levels [172]. The elevated CCL2 levels in adipose tissue and blood were also observed in humans during obesity [34], and in high-fat diet-induced [35–37] or genetically obese rodents [103, 104]. As supported by our observations, differentiated 3 T3-L1 adipocytes cultured in vitro secret high levels of CCL2, and their co-culture with LNCaP cells significantly increased the proliferation of LNCaP cells [173]. In contrast, the restriction of caloric intake delayed PCa growth in an animal study [174].

In human studies, Platz et al. found that energy intake was positively associated with metastatic or fatal PCa in certain subsets of men in a prior prospective cohort study [175]. Huber et al. found an up-regulation of CC chemokines (CCL2, CCL3, CCL5, CCL7, CCL8, and CCL11) and their respective receptors (CCR1, CCR2, CCR3, and CCR5) in adipose tissue in obese patients [176]. The expression of CCR2, CXCR1, CXCR2 and CXCR4 are higher in human PCa tissues correlated with tumor aggressiveness [10, 54, 177, 178]. These observations further support the role of obesity-related factors in promotion of PCa progression, and underline the importance to co-target obesity in treatment of PCa and some other cancers as well.

**Fig. 2:**
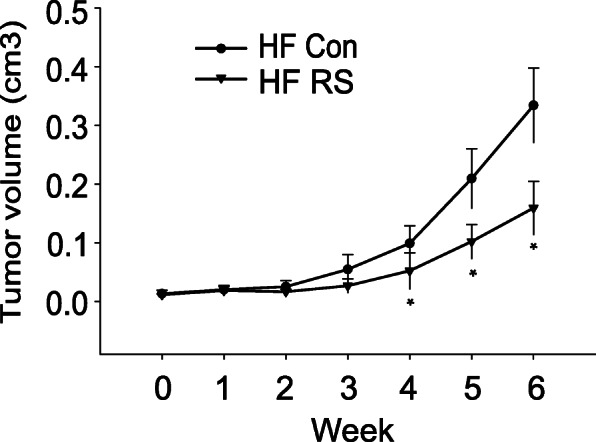
A CCR2 inhibitor RS 504393 inhibited xenograft prostate tumor growth in SCID mice fed high fat diet. Tumors were measured weekly with caliper from the initiation of treatment. Tumor volumes were calculated and data were plotted using the geometric mean for each group vs. time. Each point represents the mean tumor volume (± SD) of measurements from the 10 mice in each group

**Fig. 3:**
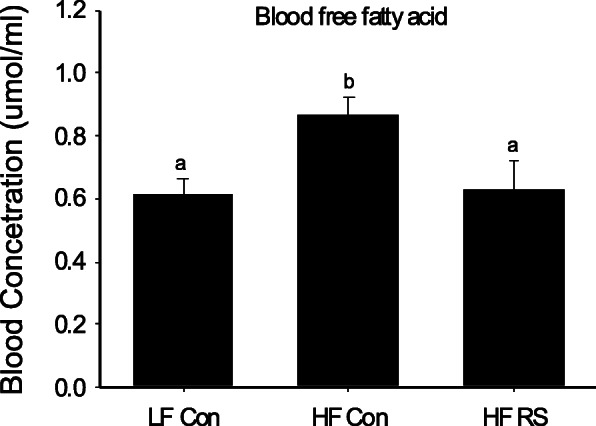
Treatment with RS 504393 reduced blood levels of free fatty acids in high-fat diet-fed SCID mice bearing LAPC-4 xenograft prostate tumor. LF Con, regular diet-fed control mice; HF Con, high-fat diet-fed control mice; HF RS, high-fat diet-fed mice treated with RS 504393. Columns with different letters indicate significant difference between groups, *P* < 0.05

We have conducted an in vivo study to assess the therapeutic potential of CCR2 inhibition in PCa in obese state. Male SCID mice were fed HFD starting 1 week prior to tumor inoculation. Mice were then implanted subcutaneously with androgen-sensitive LAPC-4 PCa cells. When tumors formed, mice were orally administered with a CCR2 inhibitor RS 504393 at 5 mg/kg body weight per day for 6 weeks. HFD-fed mice had elevated blood CCL2 levels compared to regular-diet fed mice (data not shown). Tumor growth in HFD-fed mice was significantly inhibited after 4 weeks of RS 504393 treatment, with a 50% inhibition at the end of the study (Fig. 2). Blood analysis revealed a significantly increased level of free fatty acids, a major source of fuel to cancer cells, in HFD-fed control mice compared to regular diet-fed control mice (LF Con), while the treatment with RS 504393 significantly reduced the adipocyte-release of free fatty acids in blood compared to HFD control (Fig. 3). This may suggest a novel role of CCL2/CCR2 in modulation of adipocytes’ metabolism and release of harmful factors.

**Fig. 4:**
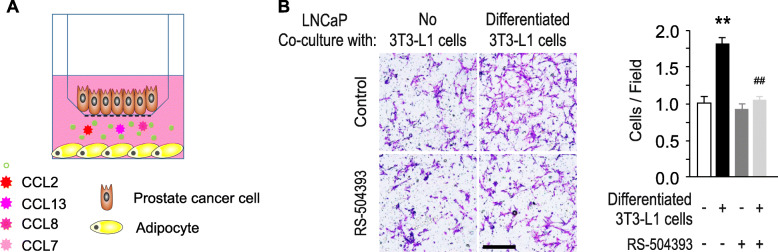
A. Schematic of LNCaP cells chemotactic assay In vitro. A. LNCaP cells migration through 8 μm pore size transwell inserts toward differentiated 3 T3-L1 cells in the bottom wells. B. Migrated LNCaP cells under a light microscope at the undersurface of the inserts were stained with crystal violet (scale bar: 20 μm). C. Cell number assessment of LNCaP cells co-cultured with differentiated 3 T3-L1 cells. **p < 0.01 versus undifferentiated 3 T3-L1 cells; ##p < 0.01 versus differentiated 3 T3-L1 cells

In addition, since CCL2-CCR2 signaling may also stimulate PCa cell migration/invasion through the layer of bone marrow endothelial cells [162], we carried out a chamber assay to test the ability of RS 504393 in inhibition of cancer cell migration in obese state (Fig. 4). Differentiated 3 T3-L1 cells were seeded on the bottom of 24-well plate. 24 h later LNCaP cells were loaded on an insert treated with or without RS 504393. Migrated cells were counted after 18 h. Co-culture with differentiated 3 T3-L1 cells significantly promotes LNCaP cell migration, while the inhibition of CCR2 signaling by RS 504393 significantly inhibited the migration of LNCaP (Fig. 4). These data provide strong support to the role of CCL2-CCR2 in prostate cancer growth and progression and indicate the therapeutic potential of this axis.

## Conclusion

Tumor progression is regulated by various intrinsic and extrinsic (microenvironment) factors. It is now well accepted that cancer cells exist in a complex environment in which they interact with a wide variety of stromal cells, including the multiple cell types that make up the immune system of the host. Many of these interactions are mediated by chemokines. The roles of chemokines in tumorigenesis have been shown to be diverse, including both negative and positive regulation of inflammatory cells, chemoattraction of tumor cells to metastatic sites, regulation of angiogenesis, and direct regulation of proliferation of cancer cells [179]. Growth factors and cytokines are supplied to PCa cells not only in an autocrine manner but also in a paracrine manner [54]. CCL2 has been shown to have direct effects on tumor growth in an autocrine and paracrine fashion in multiple cancers, including breast, lung, cervix, ovary, sarcoma, and prostate [54]. Results from our group demonstrate the therapeutic potential of CCR2 as a novel target in treatment of PCa, and possibly other types of cancer, particularly in obese state with a host CCL2-stimulated environment. CCR2 is also the receptor of CCL2, CCL13, CCL8 and CCL7, while with the highest affinity to CCL2. Evidence indicates clearly an important role of CCL2-CCR2 axis in the development and progression of PCa, possibly through both regulating monocyte/macrophage infiltration into prostate tumors and directly stimulating PCa cells.

**Fig. 5:**
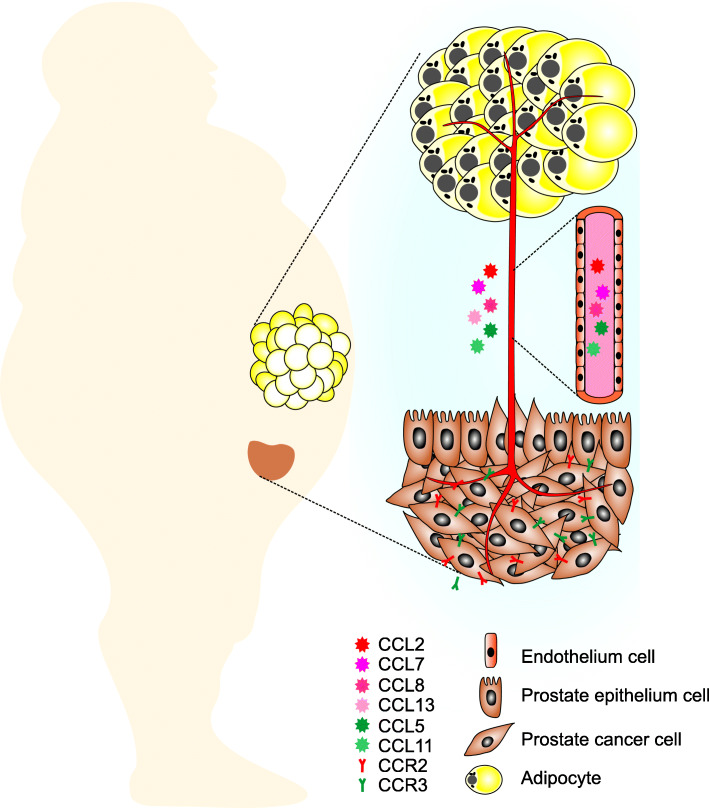
Interaction between obesity and tumor in promotion of tumor growth. During obesity, White Adipose Tissue WAT releases a plethora of molecules with autocrine, paracrine and endocrine functions including growth factors, adipokines, proinflammatory molecules, fatty acids (FA) and lipid metabolites and many others, which create a favorable condition for prostate cancer to develop

In summary, the cooperation between tumor-derived chemokines and host/adipose tissue-derived chemokines, particularly CCL2, through CCR2 signaling considerably contributes to tumor cell survival, proliferation, and metastasis (Fig. 5), which makes CCR2 a potential therapeutic target in cancer treatment. Further work is required to delineate the roles of host-derived CCL2 and tumor-derived CCL2 in PCa tumorigenesis and metastasis, and to elucidate the downstream signaling molecules which mediate the effect of CCR2 signaling in tumor promotion.

## Data Availability

The dataset(s) supporting the findings of this study are included within the article.

## Abbreviations

PCa: Prostate Cancer
CRC: Human Colorectal Cancer
CCL2: chemokine (C-C motif) Ligand 2
MCP-1: Monocyte Chemoattractant Protein-1
CCR2: CC chemokine Receptor 2
IL-8: Interleukin-8
CXCL1: Chemokine (C-X-C motif) Ligand 1
TAMs: Tumor Associated Macrophages
MDSC: Myeloid-Derived Suppressor Cells
DCs: Dendritic Cells
NK cells: Natural Killer Cells
PPAT: PeriProstatic Adipose Tissue
SVF: Stromal Vascular Fraction
STAT3: Signal Transducer and Activator of Transcription 3
BMI: Body Mass Index
HFD: High Fat Diet
TRAMP: TRansgenic Adenocarcinoma of Mouse Prostate
SCID: Severe Combined Immunodeficiency
GM-CSF: Granulocyte-macrophage colony-stimulating factor
M-CSF: Macrophage colony-stimulating factor
TNF: Tumor Necrosis Factor
